# Supplementary material for: Effect of exclusive breastfeeding cessation time on childhood morbidity and adverse nutritional outcomes in Ethiopia: Analysis of the demographic and health surveys
Source: PLoS One. 2019 Oct 2;14(10):e0223379. doi: 10.1371/journal.pone.0223379 (PMC6774524; doi:10.1371/journal.pone.0223379)
Supplement: S2 Table — (PDF) [file pone.0223379.s002.pdf]

S2 table: Explanatory variables categorization and coding

**Variables**	**Category**
Maternal age at delivery	1. <=18<br>2. 19-34<br>3. >=35
Place of residence	1. urban<br>2. rural
Number of antenatal care visits	0. No visit<br>1. 1-4 visits<br>2. >4 visits
Place of delivery	0. home<br>1. health institution
Wealth index quintile	1. poorest<br>2. poorer<br>3. middle<br>4. richer<br>5. richest
Maternal education	0. no education<br>1. primary<br>2. secondary<br>3. higher
Sex of child	1. male<br>2. female

Type of cooking fuel	1. clean fuel<br><br>2. solid fuel
Sanitation facility	0. unimproved sanitation<br><br>1. improved sanitation
Source of drinking water	1. improved<br><br>2. non-improved
Disposal of child's stools when not using toilet	1. properly disposed<br><br>2. not properly disposed
